# Effect of Benzalkonium Chloride Adaptation on Sensitivity to Antimicrobial Agents and Tolerance to Environmental Stresses in *Listeria monocytogenes*

**DOI:** 10.3389/fmicb.2018.02906

**Published:** 2018-11-28

**Authors:** Tao Yu, Xiaobing Jiang, Yige Zhang, Shengdong Ji, Wujun Gao, Lei Shi

**Affiliations:** ^1^Department of Life Science and Technology, Xinxiang University, Xinxiang, China; ^2^Department of Life Sciences, Henan Normal University, Xinxiang, China; ^3^Institute of Food Safety and Nutrition, Jinan University, Guangzhou, China

**Keywords:** *Listeria monocytogenes*, benzalkonium chloride, cross-adaptation, antibiotics, environmental stress, efflux pump

## Abstract

*Listeria monocytogenes* is an important food-borne pathogen that can persist in food processing environments and thus contaminate food products. Benzalkonium chloride (BC) is a common disinfectant widely used in food industry. Selective pressure associated with exposure to BC may result in adaptation to this agent in *L. monocytogenes*. In this study, the effect of BC adaptation on susceptibility to antimicrobial agents and tolerance to environmental stresses, as well as the role of efflux pumps in BC adaptation were investigated in *Listeria monocytogenes*. Exposure of *L. monocytogenes* to progressively increasing concentrations of BC led to adaptation not only to BC but also to several other antimicrobial agents with different modes of action, including cefotaxime, cephalothin, ciprofloxacin, and ethidium bromide (EtBr), indicating that the disinfectant BC has the ability to select for antibiotic resistance. Reserpine, an efflux pump inhibitor, reduced minimum inhibitory concentrations (MICs) of cephalosporins, ciprofloxacin, and EtBr in BC adapted strains, indicating that efflux pumps are involved in cross-adaptation to these antimicrobial agents. Our results showed that expression levels of the efflux pump MdrL in the BC adapted strains increased significantly relative to the corresponding wild-type strains (*P* < 0.05), with the highest increase in one BC adapted strain named HL06BCA. Moreover, the knockout mutant HL06BCAΔ*mdrL* showed impaired growth compared to that of HL06BCA when exposed to 2 μg/ml of BC. It suggests that efflux pump MdrL is associated with BC adaptation in *L. monocytogenes*. However, we did not find *mdrL* to be associated with cross-adaptation to cephalosporins, ciprofloxacin, and EtBr in HL06BCA. Additionally, increased sensitivity to acid, alkali, osmotic, ethanol, and oxidative stresses was observed in most strains after repeated exposure to BC. These results suggest rotation of different disinfectant is helpful to maintain high effectiveness of BC toward *L. monocytogenes* and ethanol and hydrogen peroxide are at least the appropriate candidates.

## Introduction

*Listeria monocytogenes* is a facultative intracellular, non-spore forming Gram positive bacterium which causes the severe disease listeriosis which can lead to meningitis, septicemia and mononucleosis. *L. monocytogenes* is recognized as an important foodborne pathogen and it can contaminate a variety of foods including raw foods and processed foods (Allen et al., 2016). The main contamination route for *L. monocytogenes* is through cross-contamination from production environments to food during processing (Thévenot et al., 2006; Carpentier and Cerf, 2011).

Quaternary ammonium compounds (QACs) such as benzalkonium chloride (BC) are extensively used in food processing environment to ensure the microbiological safety of food products (Jiang et al., 2016; Martínez-Suárez et al., 2016). However, inappropriate use of QACs, such as insufficient rinsing after disinfection and inadequate dosage, may lead to niches with sub-inhibitory concentrations of these compounds (Martínez-Suárez et al., 2016; Møretr et al., 2017). Adaptation to QACs develops when bacteria are frequently exposed to sub-inhibitory concentrations of QACs (Martínez-Suárez et al., 2016; Møretr et al., 2017). *L. monocytogenes* has been shown to adapt to QACs in many previous studies (Aase et al., 2000; To et al., 2002; Lundén et al., 2003; Romanova et al., 2006). This adaptation could result in increased survival of this microorganism in food environments and reduce the efficiency of QACs.

Adaptation to QACs in *L. monocytogenes* may lead to cross-adaptation to other disinfectants including tertiary alkylamine and sodium hypochlorite (Lundén et al., 2003). Moreover, cross-adaptation to antimicrobial agents with different modes of action, such as gentamicin, kanamycin, and the intercalating dye ethidium bromide (EtBr), has been found in BC adapted strains of *L. monocytogenes* (Aase et al., 2000; Romanova et al., 2006). However, our understanding of the mechanisms underlying BC adaptation in *L. monocytogenes* remains limited. So far, efflux pumps have been recognized as an important mechanism for BC adaptation in *L. monocytogenes* (Aase et al., 2000; Romanova et al., 2006; Rakic-Martinez et al., 2011). Although there are more than 200 efflux pumps in *L. monocytogenes* EGD-e predicted by Transport DB (http://www.membranetransport.org/), only a few efflux pumps have been described (Godreuil et al., 2003; Romanova et al., 2006). The role of known efflux pumps such as MdrL and Lde in adaptation to BC and cross-adaptation to other antimicrobial agents was not reported.

*L. monocytogenes* possesses the ability to overcome extreme stress conditions in food industry, such as low temperature, acids, alkaline, and high concentrations of NaCl, representing a serious threat for food safety. Previous studies reported that *L. monocytogenes* adapted to pH 4.5-5.0 had increased resistance to H_2_O_2_ and ethanol (Lou and Yousef, 1997). It also has been found that exposure to osmotic (12% NaCl), acid (pH 5), and cold (10°C) stresses resulted in increased resistance to antibiotics in *L. monocytogenes* (Al-Nabulsi et al., 2015). However, little information was available on whether QAC adaptation in *L. monocytogenes* resulted in broad resistance to different environmental stresses. Acid, alkali, osmotic, ethanol, and oxidative stresses are widely used in preserving food products or in sanitizing food processing environments. Therefore, it is necessary to assess the effect of BC adaptation on tolerance to these stresses in *L. monocytogenes*.

The aims of our study were to investigate (i) the effect of BC adaptation on susceptibility to antimicrobial agents (antibiotics, nisin, and EtBr); (ii) the role of efflux pumps in adaptation to BC and cross-adaptation to other antimicrobial agents; (iii) the effect of cephalosporin adaptation on sensitivity to BC, nisin, and EtBr; and (iv) the effect of BC adaptation on tolerance to environmental stresses in food industry (acid, alkali, osmotic, ethanol, and oxidative stresses) in *L. monocytogenes*.

## Materials and methods

### Bacterial strains and growth condition

A total of 25 strains of *L. monocytogenes* were used in this study (Table S1). Of these, 19 strains were recovered from cooked meat products, raw meat, and vegetables between August 2007 and September 2009 in Henan province. Their serotypes, PFGE patterns, and sensitivity to BC have been reported in our previous studies (Yu and Jiang, 2014; Jiang et al., 2016). An additional six strains from raw meat (two strains isolated from raw chicken meat), vegetable (one strain isolated from raw vegetable), and food production environments (three strains isolated from cooked meat production environments) were also included. Isolation of the six strains was performed using standard procedures described in the National Standards of the People's Republic of China (GB/T 4789.30-2003). Strain serotypes were assigned by the slide agglutination test using the commercial set of *Listeria* O and H antisera (Denka Seiken, Tokyo, Japan) according to the manufacturer's instructions. All the 25 strains showed lower minimum inhibitory concentrations (MICs) for BC and they were considered as BC sensitive strains according to the BC resistant breakpoint for *L. monocytogenes* (MIC ≥ 12 μg/ml) described in our previous study (Jiang et al., 2016). *L. monocytogenes* strains were grown at 37°C on brain heart infusion (BHI; Becton Dickinson, Sparks, Maryland, USA) agar or in BHI broth.

### Determination of MICs

The MICs for BC, nisin, EtBr and antibiotics against *L. monocytogenes* strains were determined using the broth microdilution method (Romanova et al., 2006). BC was purchased from Aladdin Biochemical Technology Co., Ltd. (Shanghai, China), nisin from Sigma-Aldrich (St. Louis, MO, USA; it contains 2.5% nisin with the rest NaCl and dissolved milk solids) and EtBr solution (10 mg/ml) from Takara (Tokyo, Japan). Antibiotics used in this study included ampicillin, cefotaxime, cephalothin, chloramphenicol, ciprofloxacin, erythromycin, kanamycin, and tetracycline. All antibiotics were purchased from Sigma-Aldrich. A stock solution of BC (4 mg/ml) was prepared by dissolving 0.02 g of BC in 5 ml of sterile distilled water. To prepare a nisin stock solution (1.25 mg/ml), 0.1 g of a 2.5% nisin powder was dissolved in 2 ml of 0.01 M HCl. Antibiotics stock solutions were prepared according to the manufacturer's instructions. All the stock solutions were filtered through 0.2 μm-pore-size filters (Whatman International Ltd., Maidstone, United Kingdom). For BC, the concentrations were tested from 2 to 18 μg/ml with 2 μg/ml steps. For nisin, the concentration range was 2.5–30 μg/ml with 2.5 μg/ml steps. For EtBr and all antibiotics, 2-fold serial concentrations were tested in this study. The concentration range of EtBr was 12.5–200 μg/ml. Cefotaxime and cephalothin were tested in range of 1–64 μg/ml and the other antibiotics in range of 0.0625–16 μg/ml. Strains were tested in BHI broth using 96-well microtiter plates, with an inoculum of 10^4^-10^5^ CFU/ml according to the standardized inoculum recommended by Clinical and Laboratory Standards Institute (CLSI, 2012). Growth was recorded after 18–24 h of incubation at 37°C by measuring OD at 630 nm using microplate reader (Sanco Instrument Co. Ltd., Shanghai, China). The lowest concentration of drugs totally preventing growth was taken to be the MIC. Each of the tests was done in triplicate. Interpretation for susceptibility status of 8 antibiotics used in this study was based on the standards of CLSI (2012).

### Adaptation to BC and cefotaxime

Adaptation to BC of *L. monocytogenes* was performed as described previously (Aase et al., 2000). Briefly, strains were serially subcultivated in BHI containing progressively higher BC concentrations at 37°C with shaking. Subcultivation started at a concentration of 0.5 × MIC for each strain. Subcultures were made as soon as growth had been recorded, increasing the BC concentration by steps of 0.5 μg/ml, until there was no growth within 7 days. Each BC adapted strain carried the wild-type strain designation followed by BCA. Adaptation to cefotaxime was also tested according to the same protocol as with BC adaptation. Each cefotaxime adapted strain carried the wild-type strain designation followed by CTXA.

### Efflux pumps inhibition test by using reserpine

To assess the contribution of efflux pump activity in BC adapted strains of *L. monocytogenes*, MICs of BC, EtBr, ciprofloxacin, cefotaxime, and cephalothin were examined in the presence of the efflux inhibitor reserpine (final concentration, 20 μg/mL; Sigma-Aldrich). Experiments were repeated on three separate occasions. Control cells were grown in the presence of reserpine without antimicrobial agent. This was performed to confirm that reserpine did not have an inhibitory effect on cell growth.

### RT-qPCR

Relative expression levels of *mdrL* and *lde* genes were assessed by RT-qPCR using the primers in Table S2. Total RNA was harvested from 2 ml of culture using RNAprep pure Cell/Bacteria kit (Tiangen Biotech, Beijing, China) according to the manufacturer's instruction. RNA was retro-transcribed using TIANScript RT kit (Tiangen) in a final volume of 20 μl. The PCR mix consisted of SuperReal PreMix Plus (Tiangen), specific primer pairs, and cDNA template. Amplification was performed in the LightCycler 96 real-Time PCR system (Roche, Basel, Switzerland). The PCR program was 95°C for 5 min; 40 cycles of 95°C for 30 s, 53°C for 30 s, and 72°C for 20 s. As a final step, melting curve analysis was performed between 65° and 95°C. 16S rRNA was used as an internal control for normalization in each sample. Relative transcription levels were quantified using the 2^−ΔΔ*CT*^ method (Manuel et al., 2015) and the results were shown as fold changes of the gene tested in the BC adapted strain compared to that in its wild-type strain. All experiments were performed in triplicate.

### Construction of gene deletion mutants

Strains and plasmids used for construction of gene deletion mutants are presented in Table 1. Gene deletion was performed by homologous recombination strategy, using the temperature-sensitive pMAD shuttle vector (Arnaud et al., 2004). An insert containing homologous arms up- and down-stream of the target gene was obtained by the splicing by overlap extension (SOE) PCR using the primers in Table S2. The insert and pMAD were digested using appropriate restriction enzymes (Takara) and ligated into pMAD by using T4 ligase (Takara). The recombinant plasmid was transformed into chemically competent *E. coli* DH5α cells (Biomed, Beijing, China). After confirmation by sequencing, the recombinant vector was electroporated into the competent *L. monocytogenes* cells (1.8 kV, 25 μF, 200 Ω). Transformants were selected on BHI agar plates containing erythromycin (5 μg/ml). Single-crossover mutant was selected at 39°C with erythromycin to promote chromosomal integration and double-crossover mutant at 39°C without antibiotic to enable plasmid curing. The deletions were confirmed by PCR and sequencing.

**Table 1:** Strains and plasmids used for the construction of gene deletion mutants and complementation in this study.

**Strain or plasmid**	**Genotype**	**References or source**
**STRAINS**		
***L. monocytogenes*** **strains**		
HL06	Wild-type strain; serotype 1/2c	(Yu and Jiang, 2014)
HL06BCA	BC adapted strain of HL06	This study
HL06BCAΔ*mdrL*	HL06BCA with deletion of *mdrL*	This study
HL06BCAΔ*lde*	HL06BCA with deletion of *lde*	This study
HL06Δ*mdrL*	HL06 with deletion of *mdrL*	This study
HL06Δ*lde*	HL06 with deletion of *lde*	This study
HL06Δ*mdrL*BCA	BC adapted strain of HL06Δ*mdrL*	This study
HL06Δ*lde*BCA	BC adapted strain of HL06Δ*lde*	This study
CHL06BCAΔ*mdrL*	Complemented strain of HL06BCAΔ*mdrL*	This study
HL06BCAΔ*mdrL*pERL3	HL06BCAΔ*mdrL* containing pERL3	This study
***E. coli*** **strains**		
DH5α	Chemical competent strain	Biomed, Beijing, China
DH10β	Chemical competent strain	Biomed, Beijing, China
**PLASMIDS**		
pMAD	Cloning shuttle integration vector plasmid	(Arnaud et al., 2004)
pERL3	Plasmid for complementation	(Sibelius et al., 1996)
p*mdrLc*	pERL3 containing 1,389 bp of upstream nucleotides and coding sequence of *mdrL*	This study

### Complementation of *mdrL* deletion mutant

To complement *L. monocytogenes* HL06BCAΔ*mdrL*, the complete *mdrL* open reading frame (ORF) along with its promoter was amplified from genomic DNA. After digestion with *Sac*I and *BamH*I, the PCR product was cloned into pERL3, a plasmid capable of replication in Gram-positive bacteria (Sibelius et al., 1996). The recombinant plasmid was transformed into chemically competent *E. coli* DH10β cells (Biomed) and then was electroporated into the *L. monocytogenes* HL06BCAΔ*mdrL* strain. Transformants were selected on BHI plates with erythromycin (5 μg/ml) and the presence of *mdrL* was confirmed by PCR using primers *mdrL*-7 and *mdrL*-8 (Table S2). The complemented strain was designated as CHL06BCAΔ*mdrL*. The vector control HL06BCAΔ*mdrL*pERL3 was also constructed as described above.

### Growth curve analysis

Growth curve analysis of *L. monocytogenes* were carried out as previously described (Pöntinen et al., 2015). Five colonies of each strain were individually inoculated into 5 ml of BHI broth and incubated overnight at 37°C. The cultures from HL06BCA, its deletion mutants, and complemented strain were diluted in fresh BHI broth (1:100) supplemented with BC (2 μg/ml). All the wild-type and BC adapted strains were exposed to each stress factor separately as follows. Bacteria culture were diluted in BHI broth adjusted to pH 5.5 (HCl 37%) or 9.5 (5 M NaOH) or supplemented with 6% NaCl or 1 mM H_2_O_2_ (30%) or 3.5 vol% ethanol (99.5%). Three hundred microliters of each suspension were transferred to 100-well plate. The strains were grown in a Bioscreen C microbiology reader (Growth Curves, Helsinki, Finland) at 37°C. HL06BCA, its deletion mutants, and complemented strain were grown for 24 h in 2 μg/ml BC. All the wild-type and BC adapted strains were grown for 48 h in 6% NaCl and 3.5% ethanol and 24 h for the other stress conditions. The OD_600_ was measured at 15-min intervals under each stress. The lag-phase duration, mean maximum growth rate, and maximum optical density of each strain were obtained using the DMFit program (ComBase; Computational Microbiology Research Group, Institute of Food Research, Colney, Norwich, United Kingdom), based on the models of Baranyi and Roberts. Correspondence between the OD_600_ values and viable cell numbers for the parent strain HL06BCA and each deletion mutant strain was examined by plate counts in the early logarithmic, late logarithmic, and early stationary growth phases. The area under curve (AUC) values were calculated using the software of Origin 8.0 (OriginLab Corporation, Massachusetts, USA).

### Accumulation and efflux of EtBr

The efflux of EtBr was carried out as previously described (Couto et al., 2008; Viveiros et al., 2008; Paixão et al., 2009). A LightCycler 96 instrument (Roche) was applied to obtain the fluorescence of EtBr with the excitation and emission wavelengths of 533 and 572 nm respectively. For the accumulation of EtBr, *L. monocytogenes* were grown in BHI broth to an OD_600_ of 0.6, centrifuged and washed twice in PBS. Then the suspension was adjusted to 0.3 using PBS. The cultures were incubated with 10 μg/ml EtBr (less than 1/2 the MICs of EtBr for HL06 and HL06BCA) and 20 μg/ml reserpine at 25°C for a 60 min period. For the efflux assay, *L. monocytogenes* were loaded with EtBr under conditions that favor accumulation (25°C and presence of reserpine). When the maximum level of EtBr accumulation was reached within 60 min, the bacteria were centrifuged and the broth was replaced by: (i) PBS with glucose; (ii) PBS with glucose and reserpine; and (iii) PBS containing reserpine (control of minimum efflux). The assay was performed at 37°C for a 15 min period. The efflux of EtBr is presented in terms of relative fluorescence, which is obtained from the comparison between the fluorescence value observed at each point and the control of minimum efflux.

### Statistical analysis

The statistical significance of the differences between the wild-type strains and their corresponding BC adapted strains in AUC values and maximum growth rates was tested using independent samples 2-tailed *t*-test (SPSS Statistics 23, IBM, Armonk, NY).

## Results

### MICs of BC, nisin, and EtBr for the wild-type and BC adapted strains

MICs of BC, nisin, and EtBr for the wild-type and BC adapted strains of *L. monocytogenes* are presented in Table 2. Increased BC MICs were observed in all the BC adapted strains compared to their corresponding parent strains. However, all the BC adapted strains showed MICs of nisin consistent with their corresponding parent strains. Among the 25 wild-type strains of *L. monocytogenes*, 19 and 6 strains had MICs of EtBr with 25 μg/ml and 10 μg/ml, respectively. For the BC adapted strains, 18 strains had EtBr MICs of 200 μg/ml, 5 strains of >200 μg/ml, and 2 strains of 100 μg/ml.

**Table 2:** MICs of BC, nisin, EtBr, and antibiotics for the wild-type and BC adapted strains of *L. monocytogenes*.

**Strain^a^**	**MIC (**μ**g/ml)**										
	**BC**	**Nisin**	**EtBr**	**Ampicillin**	**Cefotaxime**	**Cephalothin**	**Chloramphenicol**	**Ciprofloxacin**	**Erythromycin**	**Kanamycin**	**Tetracycline**
HL11	6	12.5	25	1	8	8	4	0.5	0.125	2	0.5
HL11BCA	12	12.5	200	1	64	32	4	1	0.125	2	0.5
HL15	6	7.5	25	0.5	2	2	8	2	0.5	2	1
HL15BCA	12	7.5	200	0.5	16	16	8	4	0.5	2	1
S7-48	6	12.5	25	0.25	8	4	8	1	0.125	1	0.5
S7-48BCA	12	12.5	200	0.25	16	16	8	2	0.125	1	0.5
HL35	2	15	25	0.5	8	8	4	1	0.125	1	1
HL35BCA	10	15	200	0.5	16	16	4	2	0.125	1	1
HL79	4	15	25	0.5	8	8	8	1	0.125	1	1
HL79BCA	10	15	200	0.5	64	64	8	4	0.125	1	1
HL95	6	12.5	10	0.25	2	2	8	1	0.125	2	1
HL95BCA	12	12.5	200	0.25	8	16	8	4	0.125	2	1
HL38	4	15	10	0.25	8	8	4	1	0.25	2	1
HL38BCA	10	15	200	0.25	64	64	4	4	0.25	2	1
HL39	6	12.5	25	0.5	8	8	4	4	0.25	1	0.5
HL39BCA	12	12.5	200	0.5	16	16	4	8	0.25	1	0.5
HL12	6	12.5	25	2	16	16	8	4	0.25	1	1
HL12BCA	12	12.5	>200	2	64	64	8	8	0.25	1	1
HL78	2	15	25	0.125	16	16	4	1	0.125	2	1
HL78BCA	10	15	200	0.125	64	64	4	4	0.125	2	1
HL50	4	15	25	0.25	16	16	4	1	0.25	1	1
HL50BCA	10	15	>200	0.25	64	64	4	4	0.25	1	1
HL60	6	12.5	25	0.25	8	8	4	0.5	0.125	1	1
HL60BCA	12	12.5	200	0.25	16	16	4	1	0.125	1	1
HL82	6	15	25	2	32	32	8	1	0.25	1	1
HL82BCA	10	15	200	2	64	64	8	2	0.25	1	1
HL90	2	7.5	10	0.25	8	8	8	1	0.125	1	1
HL90BCA	10	7.5	200	0.25	16	16	8	2	0.125	1	1
HL17	6	15	10	1	8	8	4	1	0.125	1	1
HL17BCA	14	15	200	1	32	32	4	2	0.125	1	1
HL26	6	12.5	10	1	8	8	4	1	0.125	2	0.5
HL26BCA	12	12.5	200	1	64	64	4	2	0.125	2	0.5
HL88	6	15	25	0.5	8	8	1	1	0.5	2	0.5
HL88BCA	14	15	>200	0.5	64	64	1	2	0.5	2	0.5
HL06	6	15	25	0.5	8	8	8	0.5	0.125	1	0.5
HL06BCA	14	15	100	0.5	16	16	8	1	0.125	1	0.5
HL24	6	15	10	1	8	8	4	1	0.5	1	1
HL24BCA	14	15	200	1	32	32	4	2	0.5	1	1
HL28	4	20	25	0.5	4	4	4	0.5	0.125	4	0.5
HL28BCA	14	20	>200	0.5	32	32	4	1	0.125	4	0.5
S36-84	6	15	25	1	16	16	4	0.5	0.25	2	0.5
S36-84BCA	14	15	>200	1	32	32	4	1	0.25	2	0.5
S45-86	6	15	25	1	16	32	8	0.5	0.125	2	1
S45-86BCA	12	15	200	1	32	64	8	1	0.125	2	1
S51-88	6	12.5	25	1	16	16	8	1	0.125	1	1
S51-88BCA	12	12.5	100	1	64	64	8	2	0.125	1	1
S15-90	6	12.5	25	1	8	8	8	1	0.125	2	1
S15-90BCA	12	12.5	200	1	16	16	8	2	0.125	2	1
S1-73	6	12.5	25	1	16	16	4	1	0.125	2	1
S1-73BCA	12	12.5	200	1	64	64	4	2	0.125	2	1

a *Each BC adapted strain carried the wild-type strain designation followed by BCA*.

### MICs of antibiotics for the wild-type and BC adapted strains

MICs of 8 antibiotics for the wild-type and BC adapted strains of *L. monocytogenes* are presented in Table 2. All the BC adapted strains exhibited MICs of ampicillin, chloramphenicol, erythromycin, kanamycin and tetracycline consistent with their corresponding wild-type strains. However, increased MICs against cefotaxime, cephalothin, and ciprofloxacin were observed in all the BC adapted strains compared to their corresponding parent strains. Among the 25 wild-type strains of *L. monocytogenes*, 17 strains were sensitive (MIC ≤ 8 μg/ml) and 8 were intermediate to cefotaxime (MIC = 16 μg/ml or 32 μg/ml). After BC adaptation, 13 strains were intermediate to cefotaxime, 11 strains exhibited cefotaxime resistant (MIC ≥ 64 μg/ml), and only one strain was cefotaxime susceptible. Among the 25 wild-type strains of *L. monocytogenes*, 17 strains were sensitive (MIC ≤ 8 μg/ml), 6 were intermediate (MIC = 16 μg/ml), and 2 were resistant to cephalothin (MIC ≥ 32 μg/ml). After BC adaptation, 16 strains exhibited resistance to cephalothin and 9 were intermediate to cephalothin. None of the adapted strains showed cephalothin susceptibility. Among the 25 wild-type strains of *L. monocytogenes*, 22 strains were sensitive (MIC ≤ 1 μg/ml), 1 was intermediate (MIC = 2 μg/ml), and 2 were resistant to ciprofloxacin (MIC ≥ 4 μg/ml). For the BC adapted strains, 6 strains were sensitive, 11 were intermediate, and 8 were resistant to ciprofloxacin.

### Effect of reserpine on MICs of selected antimicrobial agents for BC adapted strains

All the BC adapted strains showed reduced MICs of BC in the presence of reserpine (Table 3). However, the values were still higher than those of the corresponding wild-type strains (Table 2). Addition of reserpine resulted in decreased MICs of EtBr for all the BC adapted strains with two-fold decreases observed in most of strains (Table 3). In the presence of reserpine, all the BC adapted strains exhibited increased sensitivity to cefotaxime and cephalothin (Table 3). MICs of cefotaxime and cephalothin for most BC adapted strains in the presence of reserpine were similar to or lower than those of their wild-type strains without reserpine (Table 2). The majority of the BC adapted strains had reduced MICs of ciprofloxacin when exposed to reserpine (Table 3).

**Table 3:** MICs of selected antimicrobial agents for BC adapted strains of *L. monocytogenes* in the presence of reserpine.

**Strain**	**MIC (**μ**g/ml)**									
	**BC**	**+Reserpine**	**EtBr**	**+Reserpine**	**Cefotaxime**	**+Reserpine**	**Cephalothin**	**+Reserpine**	**Ciprofloxacin**	**+Reserpine**
HL11BCA	12	8	200	100	64	4	32	8	1	1
HL15BCA	12	8	200	100	16	4	16	4	4	1
S7-48BCA	12	8	200	100	16	4	16	4	2	1
HL35BCA	10	6	200	100	16	4	16	4	2	1
HL79BCA	10	6	200	100	64	8	64	8	4	2
HL95BCA	12	10	200	100	8	4	16	4	4	2
HL38BCA	10	6	200	100	64	8	64	8	4	1
HL39BCA	12	8	200	100	16	4	16	4	8	2
HL12BCA	12	8	>200	100	64	8	64	8	8	2
HL78BCA	10	6	200	100	64	8	64	8	4	2
HL50BCA	10	6	>200	100	64	8	64	8	4	2
HL60BCA	12	8	200	100	16	4	16	4	1	1
HL82BCA	10	6	200	100	64	8	64	8	2	1
HL90BCA	10	6	200	100	16	4	16	4	2	1
HL17BCA	14	10	200	100	32	4	32	4	2	1
HL26BCA	12	10	200	100	64	4	64	4	2	1
HL88BCA	14	10	>200	100	64	4	64	16	2	1
HL06BCA	14	10	100	50	16	4	16	4	1	1
HL24BCA	14	10	200	100	32	4	32	4	2	1
HL28BCA	14	10	>200	100	32	2	32	2	1	1
S36-84BCA	14	10	>200	100	32	4	32	4	1	1
S45-86BCA	12	10	200	100	32	4	64	4	1	1
S51-88BCA	12	8	100	50	64	32	64	32	2	1
S15-90BCA	12	8	200	100	16	4	16	4	2	1
S1-73BCA	12	8	200	100	64	8	64	8	2	1

### Relative expression levels of efflux pump genes *mdrL* and *lde*

**Figure 1:**
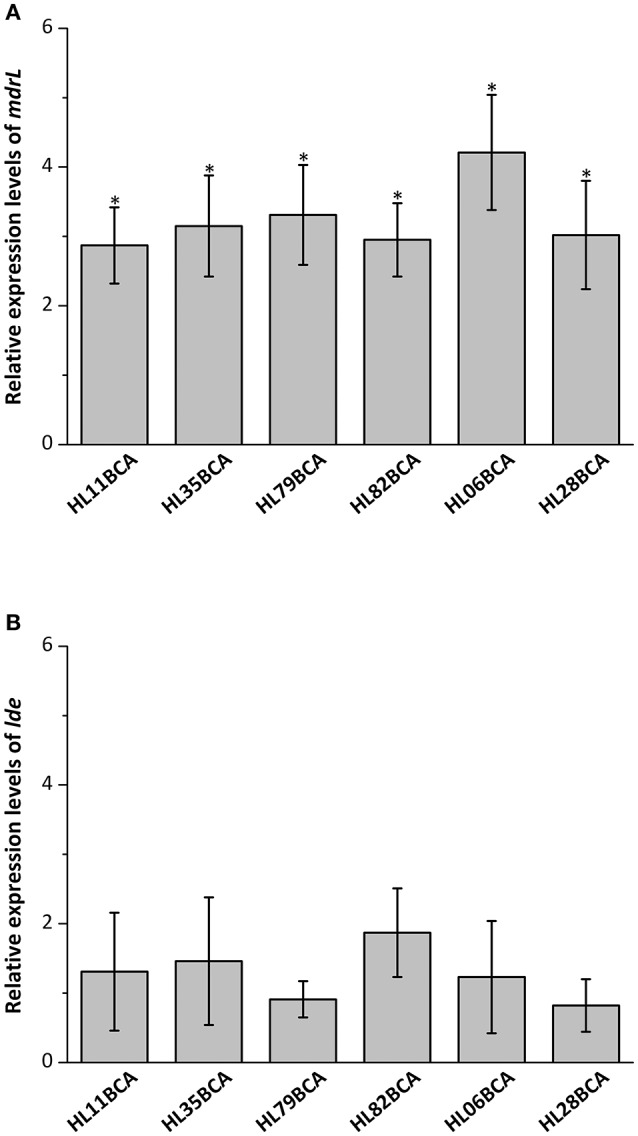
**(A)** Relative expression levels of *mdrL* in six BC adapted strains in BHI broth. **(B)** Relative expression levels of *lde* in six BC adapted strains in BHI broth. Results are presented as fold changes of the gene tested in the BC adapted strain compared to that in its wild-type strain in BHI broth. Error bars represent the standard deviation of triplicate experiments (*n* = 3). The asterisk indicates a value statistically different from that of HL06 grown in BHI, with a *P*-value < 0.05.

Six wild-type strains and their corresponding BC adapted strains were selected and the relative expression levels of *mdrL* and *lde* in these strains were investigated in our study. Results from RT-qPCR showed that expression levels of *mdrL* in the BC adapted strains increased significantly in relative to the corresponding wild-type strains (*P* < 0.05), with the highest increase in HL06BCA (Figure 1A). However, no significant difference was observed in expression level of *lde* after BC adaptation (Figure 1B).

### Phenotypes of gene deletion mutants

To clarify the role of MdrL and Lde in BC adaptation, the deletion mutant strains of *mdrL* and *lde* derived from HL06BCA were constructed in this study. The mutants HL06BCAΔ*mdrL* and HL06BCAΔ*lde* showed the same MICs of selected antimicrobial agents as their parent strain HL06BCA (Table 4). In the particular case of BC, no difference in the MICs was observed between the deletion mutant and parent strains.

**Table 4:** MICs of selected antimicrobial agents for deletion mutant strains of *L. monocytogenes*.

**Strain**	**MIC (**μ**g/ml)**				
	**BC**	**EtBr**	**Cefotaxime**	**Cephalothin**	**Ciprofloxacin**
HL06	6	25	8	8	0.5
HL06BCA	14	100	16	16	1
HL06BCAΔ*mdrL*	14	100	16	16	1
HL06BCAΔ*lde*	14	100	16	16	1
HL06Δ*mdrL*	6	25	8	8	0.5
HL06Δ*lde*	6	25	8	8	0.5
HL06Δ*mdrL*BCA	14	100	16	16	1
HL06Δ*lde*BCA	14	100	16	16	1

**Figure 2:**
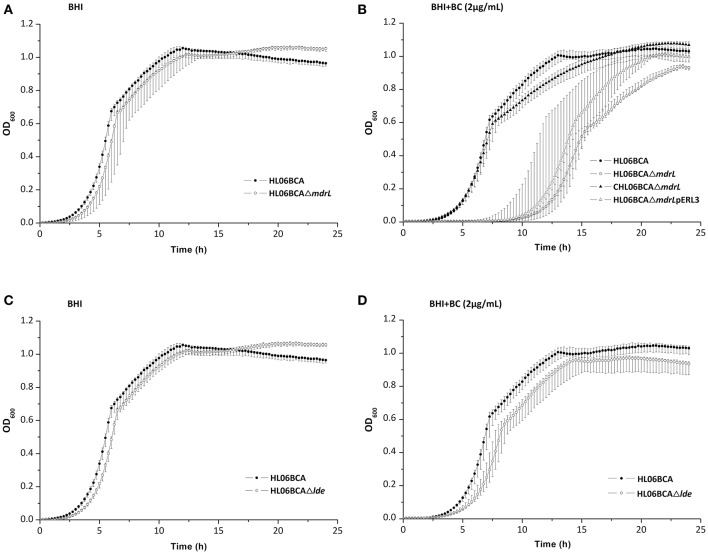
**(A)** Growth curves for *L. monocytogenes* HL06BCA and HL06BCAΔ*mdrL* in BHI broth. **(B)** Growth curves for *L. monocytogenes* HL06BCA, HL06BCAΔ*mdrL*, CHL06BCAΔ*mdrL*, and HL06BCAΔ*mdrL*pERL3 in BHI broth with 2 μg/mL of BC. **(C)** Growth curves for *L. monocytogenes* HL06BCA and HL06BCAΔ*lde* in BHI broth. **(D)** Growth curves for *L. monocytogenes* HL06BCA and HL06BCAΔ*lde* in BHI broth with 2 μg/mL of BC.

When exposed to 2 μg/ml of BC, the mutant HL06BCAΔ*mdrL* showed impaired growth compared to that of the parent strain HL06BCA (Figure 2B). A significantly longer lag-phase duration was observed for HL06BCAΔ*mdrL* than for HL06BCA (*P* < 0.0001; Table 5). The mean maximum growth rate of HL06BCAΔ*mdrL* was 29.2% (*P* < 0.001) lower and the mean maximum optical density 9.5% (*P* < 0.05) lower than those of HLBCA (Table 5). In the presence of BC, HL06BCAΔ*lde* showed a slightly longer lag-phase duration (*P* > 0.05), a 16.9% (*P* > 0.05) lower growth rate and an 8.6% (*P* < 0.05) lower maximum optical density than those of HL06BCA (Figure 2D; Table 5). In BHI medium without BC, the growth of HL06BCAΔ*mdrL* and HL06BCAΔ*lde* was similar to that of HL06BCA (Figures 2A,C). Complementation of the deletion mutant strain HL06BCAΔ*mdrL* restored the phenotype of the mutant strain in the presence of BC (2 μg/ml) to the level of HL06BCA (Figure 2B). The growth curve for the vector control was similar to that for HL06BCAΔ*mdrL* (Figure 2B).

**Table 5:** Average lag phase durations, mean maximum growth rates, and mean maximum optical densities of *L. monocytogenes* HL06BCA and its mutants HL06BCAΔ*mdrL* and HL06BCAΔ*lde* in BHI broth with and without BC.

**Growth parameter**	**Medium**	**Strain**		
		**HL06BCA**	**HL06BCAΔ*mdrL***	**HL06BCAΔ*lde***
Lag-phase duration (h)^a^	BHI	3.11 ± 0.030	3.54 ± 0.352	3.62 ± 0.101
	BHI+BC	4.00 ± 0.186	10.70 ± 0.269^****^	4.85 ± 0.472
Mean maximum growth rate ±*SD*(*OD*_600*units*/*h*)^a	BHI	0.190 ± 0.011	0.163 ± 0.016	0.170 ± 0.005
	BHI+BC	0.154 ± 0.003	0.109 ± 0.002^***^	0.128 ± 0.011
Mean maximum optical density ±*SD*(*OD*_600*units*)^a	BHI	1.04 ± 0.030	1.06 ± 0.009	1.06 ± 0.011
	BHI+BC	1.04 ± 0.028	0.94 ± 0.012^*^	0.95 ± 0.051^*^

a Significantly decreased values compared to the corresponding value of HL06BCA are indicated by asterisks (

* P < 0.05;

*** P < 0.001;

**** *P < 0.0001). P-values were obtained using independent samples 2-tailed t-test (SPSS Statistics 23)*.

The gene deletion mutants of *mdrL* and *lde* derived from the wild-type strain HL06 were also constructed in this study. Our results showed that neither absence of *mdrL* nor absence of *lde* had effect on the MICs of selected compounds for HL06 (Table 4). BC adapted strains of the deletion mutants HL06Δ*mdrL* and HL06Δ*lde*, named HL06Δ*mdrL*BCA and HL06Δ*lde*BCA, respectively, were obtained by exposure to progressively increasing concentrations of BC. Interestingly, HL06Δ*mdrL*BCA and HL06Δ*lde*BCA had the same MICs of BC and other antimicrobial agents as HL06BCA which was BC adapted strain of HL06 (Table 4).

**Figure 3:**
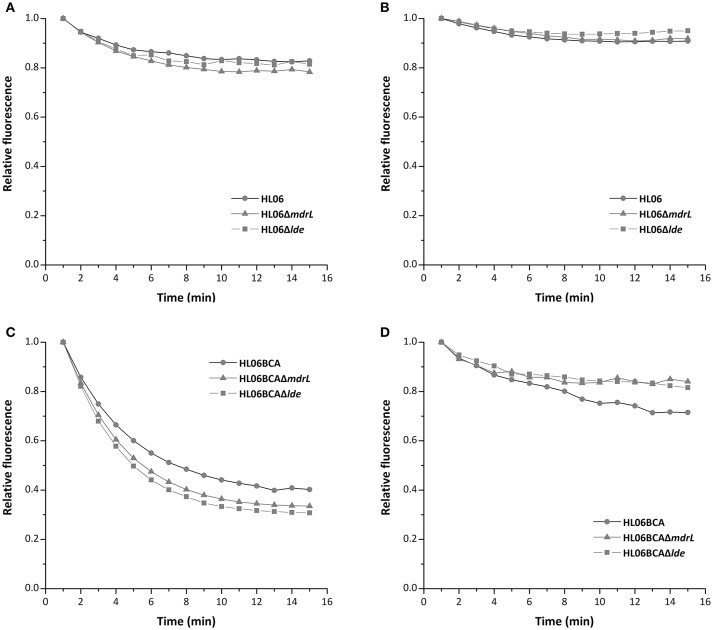
Evaluation of EtBr efflux activity for HL06, HL06Δ*mdrL*, and HL06Δ*lde* under different conditions: **(A)** In the presence of glucose without reserpine; **(B)** in the presence of glucose and reserpine. Evaluation of EtBr efflux activity for HL06BCA, HL06BCAΔ*mdrL*, and HL06BCAΔ*lde* under different conditions: **(C)** in the presence of glucose without reserpine; **(D)** in the presence of glucose and reserpine.

To investigate the efflux activity in the parent and mutant strains, glucose was used to provide energy for the extrusion of EtBr. As shown by Figure 3, efflux of EtBr from all the tested strains took place after the addition of glucose. However, no difference was observed between HL06 and its deletion mutant strains, neither between HL06BCA and its deletion mutants (Figure 3). The presence of reserpine with medium containing glucose inhibited the efflux of EtBr in all the tested stains (Figure 3).

### MICs of cefotaxime, cephalothin, BC, nisin, and EtBr for cefotaxime adapted strains

As shown in Table 6, all the cefotaxime adapted strains of *L. monocytogenes* showed increased MICs of cefotaxime and cephalothin and high-level resistance to these antibiotics. MICs of nisin for all the strains also increased after adaptation to cefotaxime. However, cefotaxime adaptation did not affect MICs of BC and EtBr.

**Table 6:** MICs of cefotaxime, BC, nisin, EtBr, and cephalothin for the wild-type and cefotaxime adapted strains of *L. monocytogenes*.

**Strain^a^**	**MIC (**μ**g/ml)**				
	**Cefotaxime**	**BC**	**Nisin**	**EtBr**	**Cephalothin**
HL11	8	6	12.5	25	8
HL11CTXA	>64	6	25	25	>64
HL15	2	6	7.5	25	2
HL15CTXA	64	6	25	25	64
S7-48	8	6	12.5	25	4
S7-48CTXA	>64	6	25	25	>64
HL35	8	2	15	25	8
HL35CTXA	>64	2	25	25	>64
HL79	8	4	15	25	8
HL79CTXA	>64	4	25	25	>64
HL95	2	6	12.5	10	2
HL95CTXA	64	6	25	10	64
HL38	8	4	15	10	8
HL38CTXA	>64	4	25	10	>64
HL39	8	6	12.5	25	8
HL39CTXA	>64	6	25	25	>64
HL12	16	6	12.5	25	16
HL12CTXA	>64	6	20	25	>64
HL78	16	2	15	25	16
HL78CTXA	>64	2	25	25	>64
HL50	16	4	15	25	16
HL50CTXA	>64	4	25	25	>64
HL60	8	6	12.5	25	8
HL60CTXA	>64	6	25	25	>64
HL82	32	6	15	25	32
HL82CTXA	>64	6	25	25	>64
HL90	8	2	7.5	10	8
HL90CTXA	64	2	20	10	64
HL17	8	6	15	10	8
HL17CTXA	>64	6	25	10	>64
HL26	8	6	12.5	10	8
HL26CTXA	>64	6	20	10	>64
HL88	8	6	15	25	8
HL88CTXA	>64	6	25	25	>64
HL06	8	6	15	25	8
HL06CTXA	>64	6	25	25	>64
HL24	8	6	15	10	8
HL24CTXA	>64	6	25	10	>64
HL28	4	4	20	25	4
HL28CTXA	>64	4	25	25	>64
S36-84	16	6	15	25	16
S36-84CTXA	64	6	25	25	64
S45-86	16	6	15	25	32
S45-86CTXA	64	6	25	25	64
S51-88	16	6	12.5	25	16
S51-88CTXA	>64	6	25	25	>64
S15-90	8	6	12.5	25	8
S15-90CTXA	>64	6	25	25	>64
S1-73	16	6	12.5	25	16
S1-73CTXA	>64	6	25	25	>64

a *Each cefotaxime adapted strain carried the wild-type strain designation followed by CTXA*.

### Growth of the wild-type and BC adapted strains under different stress conditions

**Figure 4:**
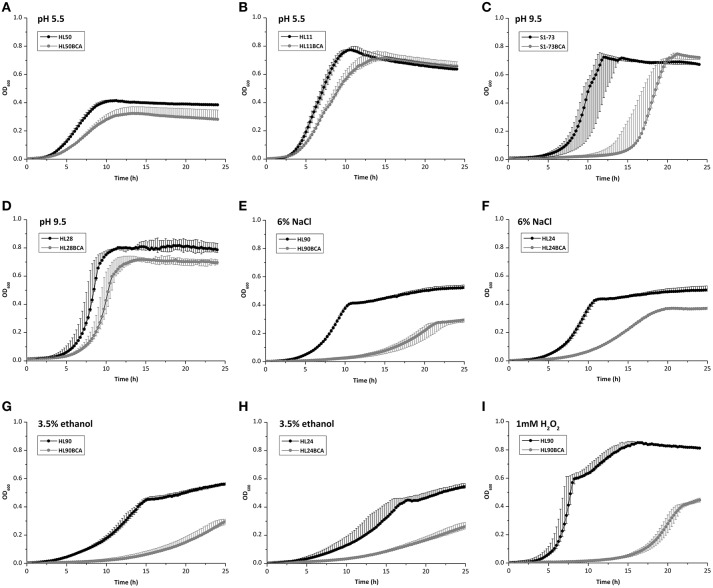
Growth curves of **(A)** HL50 and HL50BCA and **(B)** HL11 and HL11BCA under acid stress; **(C)** S1-73 and S1-73BCA and **(D)** HL28 and HL28BCA under alkali stress; **(E)** HL90 and HL90BCA and **(F)** HL24 and HL24BCA under osmotic stress; **(G)** HL90 and HL90BCA and **(H)** HL24 and HL24BCA under ethanol stress; **(I)** HL90 and HL90BCA under oxidative stress.

When exposed to acid stress, AUC values of 23 BC adapted strains decreased and 2 adapted strains (S51-88 and S1-73) increased slightly (Table S3), with the largest decrease of AUC being observed between HL50 and its BC adapted strain HL50BCA (27.0%; Figure 4A); the maximum growth rates of 22 BC adapted strains decreased and 3 adapted strains (HL26, S51-88, and S15-90) increased (Table S4), compared to the corresponding wild-type strains. The maximum growth rates of HL11 and HL11BCA presented the largest decrease with 37.7% under acid stress (Figure 4B).

Compared to the corresponding wild-type strains, all adapted strains showed decreased AUC values and decreased maximum growth rates were observed in all adapted strains except one (S1-73) under alkali stress (Tables S3, S4). AUC values of S1-73 and S1-73BCA exhibited the largest decrease with 52.7% (Figure 4C) and the maximum growth rates of HL28 and HL28BCA showed the largest decrease with 26.9% (Figure 4D) exposed to alkali stress.

In the presence of 6% NaCl, all adapted strains showed decreased AUC values and maximum growth rates in relation to corresponding wild-type strains (Tables S3, S4). The largest decrease of AUC values and the maximum rates between the wild-type strains and their adapted strains were observed in HL90 (48.4%) and HL24 (67.9%) under osmotic stress, respectively (Figures 4E,F).

In the presence of 3.5% ethanol, decreased AUC values and maximum growth rates were observed in all adapted strains compared to their corresponding wild-type strains (Tables S3, S4). When exposed to ethanol stress, HL90 and HL90BCA presented the largest decrease of AUC values (50.4%; Figure 4G); the maximum growth rates of HL24 and HL24BCA showed the largest decrease with 59.0% (Figure 4H).

When exposed to 1 mM H_2_O_2_, all adapted strains showed decreased AUC values; the maximum growth rates of 21 BC adapted strains decreased and 4 adapted strains showed increased maximum growth rates compared to corresponding wild-type strains (Tables S3, S4). HL90 and HL90BCA presented the largest decrease of AUC values (81.5%) and the maximum growth rates (53.5%) under oxidative stress (Figure 4I).

## Discussion

In our study, all the *L. monocytogenes* strains tested had the ability to develop increased tolerance to BC on regular exposure to BC. The highest increase in BC MICs was 5-fold, which was found in 3 strains with initial MICs of 2 μg/ml. Adaptive responses were greatest when the initial MICs for strains were low. This phenomenon was also reported in previous studies (Lundén et al., 2003). On the whole, all the strains reached approximately similar MICs of BC after BC adaptation, which is consistent with the earlier studies (Aase et al., 2000; Lundén et al., 2003). The mechanisms behind BC adaptation in *L. monocytogenes* were complicated. It was proposed that the possible mechanisms of BC adaptation in *L. monocytogenes* were decreased uptake or active efflux for pumping the disinfectant from the cell (Aase et al., 2000). Until now, the important role of efflux pumps in adaptation to BC has been reported in *L. monocytogenes* (Aase et al., 2000; Romanova et al., 2006; Rakic-Martinez et al., 2011). In this study, the addition of efflux pump inhibitor reserpine led to the decreased MICs of BC for all the BC adapted strains, indicating that the activity of efflux pumps in these strains was inhibited by reserpine. It also supports the view that efflux pumps contribute to BC adaptation in *L. monocytogenes* (Aase et al., 2000; Romanova et al., 2006). However, the BC MICs of BC adapted strains in the presence of reserpine were still higher than those of the corresponding wild-type strains in the absence of reserpine. These results could be explained in two ways. Given that reserpine could not exhibit good inhibition on all types of efflux pumps (Marquez, 2005), it is possible that some efflux pump(s) responsible for BC adaptation is (are) not inhibited by reserpine effectively. In addition, there is the possibility that decreased uptake of BC or other unknown mechanism is also involved in BC adaptation in these strains. Our results suggest that efflux pumps indeed play a role in BC adaptation in *L. monocytogenes*, but it is likely not the only mechanism for BC adaptation.

Overexpression of efflux pump MdrL, a multidrug resistance pump belonging to major facilitator superfamily (MFS), was considered to be partly responsible for adaptation of naturally sensitive strains of *L. monocytogenes* to BC (Romanova et al., 2006). For six wild-type strains tested in our study, the relative expression levels of *mdrL* were significantly higher after adaptation to BC, indicating that MdrL could be involved in BC adaptation in *L. monocytogenes*. The highest increase was observed between HL06 and HL06BCA. To further investigate the role of MdrL in BC adaptation, the gene deletion mutants HL06Δ*mdrL* and HL06BCAΔ*mdrL* were constructed. In the presence of BC, the growth of HL06BCAΔ*mdrL* was impaired in relation to the parent strain HL06BCA. The growth of HL06BCAΔ*mdrL* was similar to that of HL06BCA in the absence of BC, suggesting that *mdrL* is associated with BC adaptation of HL06. The restored phenotype of the complemented strain CHL06BCAΔ*mdrL* confirmed the growth defect of HL06BCAΔ*mdrL* to be due specifically to the deletion of *mdrL*. On the other hand, the mutant HL06Δ*mdrL* could also adapt to BC by increasing concentrations of BC and its BC adapted strain which was named HL06Δ*mdrL*BCA showed the same MIC of BC to that of HL06BCA (the BC adapted strain of HL06). It suggests that other efflux pump(s), besides MdrL, are also involved in BC adaptation in *L. monocytogenes*.

Another MFS-type efflux pump Lde was also investigated in this study. For six wild-type strains tested in our study, no significant difference was observed in expression levels of *lde* after BC adaptation, which was consistent with the previous findings (Romanova et al., 2006). The growth of the deletion mutant HL06BCAΔ*lde* was similar to that of HL06BCA in the presence or absence of BC. After BC adaptation, the mutant HL06Δ*lde* exhibited the same MIC of BC as HL06BCA. Our results indicate that efflux pump Lde is not associated with BC adaptation in *L. monocytogenes*.

Our results showed that BC adapted strains of *L. monocytogenes* had increased MICs of EtBr, suggesting cross-adaptation between BC and EtBr in *L. monocytogenes*. Similar observations were also found in previous studies (Aase et al., 2000; Romanova et al., 2006). BC and EtBr are found to be substrates for the same efflux pump in *Staphylococcus*, although they are structurally different (Heir et al., 1998, 1999). In this study, decreased MICs of EtBr were also observed for all the BC adapted stains in the presence of reserpine, supporting the previous findings that cross-adaptation to BC and EtBr was attributed to efflux pumps (Aase et al., 2000).

Interestingly, increased MICs of cefotaxime and cephalothin were found in all the BC adapted strains of *L. monocytogenes* in the present study. And more importantly, most originally sensitive or intermediate strains became resistance to cefotaxime and cephalothin after BC adaptation. Cross-resistance to cephalosporins in *L. monocytogenes* adapted to BC implied that a common mechanism was likely involved. It has been shown that efflux pumps contribute to cephalosporins resistance in *L. monocytogenes* (Collins et al., 2010a). Results from efflux inhibition testing showed the reduced MICs of cefotaxime and cephalothin for all the BC adapted strains after the addition of reserpine. Moreover, these MICs were similar to or lower than those of the corresponding wild-type strains in the absence of reserpine. Therefore, enhanced efflux appears to be an important mechanism for the observed cross-resistance to cephalosporins in BC adapted strains of *L. monocytogenes*.

Mata et al. (2000) found that the allele-substituted mutant of *mdrL* presented lower MICs of cefotaxime and failed to pump out EtBr. However, both mutants HL06Δ*mdrL* and HL06BCAΔ*mdrL* showed the consistent MICs of cefotaxime and EtBr that were not affected by deletion of *mdrL* in our study. Furthermore, the decreased capacity to extrude EtBr was not observed in HL06Δ*mdrL* and HL06BCAΔ*mdrL*, suggesting that efflux pump MdrL was not involved in cross-resistance to EtBr and cephalosporins in *L. monocytogenes*. Godreuil et al. (2003) have reported that efflux pump Lde could be partly responsible for EtBr resistance in *L. monocytogenes*. But our results demonstrated that Lde was not associated with cross-resistance to EtBr, at least in HL06BCA. There is the possibility that cross-resistance to cefotaxime, cephalothin, and EtBr in *L. monocytogenes* is due to other efflux pumps which are not yet identified or other unknown mechanisms.

Effect of cefotaxime adaptation on sensitivity to BC, nisin, and EtBr in *L. monocytogenes* was also investigated in this study. As observed, cross-adaptation to cephalothin with a similar mode of action and nisin with a different mode of action occurred after adaptation to cefotaxime. Similarly, cross-adaptation to cefotaxime and nisin was observed in cephalothin adapted strains of *L. monocytogenes* (data not shown). Nisin is a ribosomally synthesized cationic antimicrobial peptide that is used in the preservation of food for a long time (Crandall and Montville, 1998). Recent studies have shown that nisin resistance is linked to cephalosporins resistance, as several genes play roles in both cephalosporins and nisin resistance ((Cotter et al., 2002; Collins et al., 2010a,b); (Collins et al., 2012)). Therefore, it was not surprising that cross-adaptation to nisin was found in cefotaxime and cephalothin adapted strains of *L. monocytogenes* in our study. Notably, BC adaptation didn't cause adaptation to nisin, however, cross-adaptation to nisin was observed in cefotaxime adapted strains of *L. monocytogenes*. Given that nisin resistance and cephalosporins resistance have been found to be linked, cross-adaptation between cephalosporins (cefotaxime and cephalothin) and nisin is likely due to a shared specific mechanism of action.

Besides cephalosporins, fluorquinolones are most frequently applied in clinical settings or environments. In this study, cross-resistance to ciprofloxacin was also found in *L. monocytogenes* adapted to BC, which was in agreement with previous findings (Rakic-Martinez et al., 2011). Generally, the main mechanisms behind ciprofloxacin resistance are mutation(s) in quinolone resistance determining regions (QRDRs) of DNA gyrase and topoisomerase IV and active efflux (Hernández et al., 2011). Mutation in QRDRs was not observed in any of BC adapted strains (data not shown). The role of efflux pumps in ciprofloxacin resistance in *L. monocytogenes* has been confirmed (Godreuil et al., 2003; Jiang et al., 2012; Guérin et al., 2014). Our study found that MICs of ciprofloxacin for most of the BC adapted strains decreased in the presence of reserpine, supporting that efflux pumps are involved in cross-adaptation to ciprofloxacin in BC adapted strains of *L. monocytogenes*. Although all the strains showed increased MICs of ciprofloxacin after BC adaptation, only a few strains could be categorized as resistant according to their final MICs of ciprofloxacin. This suggests that efflux pumps could be associated with low-level resistance to ciprofloxacin. Previously, Godreuil et al. (2003) reported that efflux pump Lde was associated with ciprofloxacin resistance in *L. monocytogenes*. In our previous works, overexpression of *lde* was observed in ciprofloxacin resistant strains of *L. monocytogenes* when exposed to sublethal concentration of ciprofloxacin (Jiang et al., 2012). In the current study, adaptation to BC did not increase the expression level of *lde*. Lacking *lde* had no effect on the MIC of ciprofloxacin in HL06BCA. Our results suggest that Lde could not be involved in cross-resistance to ciprofloxacin in BC adapted strain of *L. monocytogenes*, although this pump is found to be responsible for ciprofloxacin resistance in *L. monocytogenes* (Godreuil et al., 2003).

Our results demonstrated that BC adaptation of *L. monocytogenes* did not result in increased MICs of kanamycin. According to Romanova et al. (2006), increased MICs of kanamycin were observed in two strains after BC adaptation; however, the other two strains had the same MICs of kanamycin before and after BC adaptation. We speculated that cross-resistance of two BC adapted strains to kanamycin could be due to the unique presence of plasmids containing efflux pump genes responsible for kanamycin resistance such that BC adaptation improved the efflux activity of kanamycin, leading to increased resistance to this agent in these strains. Accordingly, absence of drug resistance efflux pump(s) located on plasmids could be the reason that cross-resistance to kanamycin did not occur in our strains and the strains from Romanova et al. (2006). Ampicillin is the first choice therapy for listeriosis, sometimes given in combination with gentamicin (Mora et al., 1998). However, cross-resistance to ampicillin was not observed in BC-adapted strains of *L. monocytogenes* (Table 2), indicating that ampicillin remains a useful treatment for listeriosis.

Acid, alkali, osmotic stresses are widely used in preserving food products, and ethanol and oxidative stresses in sanitizing food processing environments. Exposure to BC resulted in most strains in our study becoming more sensitive to the stresses mentioned above, suggesting that the combination of BC and other environmental stress such as acid could be more effective for control *L. monocytogenes* in food or food environments. The frequent use of BC can give rise to poor disinfectant effect on *L. monocytogenes* due to bacterial adaptive response. In this case, rotation of disinfecting agents with different mechanisms of action in food processing plants is beneficial in disinfecting to prevent the development of resistant strains (Lundén et al., 2003). Our results suggested that ethanol and hydrogen peroxide could be the good substitutes for BC in practical application.

## Conclusion

In summary, the present study showed cross-adaptation to cephalosporins, ciprofloxacin, and EtBr in BC adapted strains of *L. monocytogenes*, supporting the view that disinfectant has the ability to select for antibiotic resistance in bacteria. Adaptation to BC resulted in cross-adaptation to antimicrobial agents with different modes of action, suggesting that the mechanism leading to BC adaptation of *L. monocytogenes* is non-specific. Efflux pumps are involved not only in adaptation to BC in *L. monocytogenes* but also in cross-adaptation to cephalosporins, ciprofloxacin, and EtBr in BC adapted strains. Efflux pump MdrL, instead of Lde, is associated with BC adaptation in *L. monocytogenes*. However, neither MdrL nor Lde contributes to cross-adaptation to cephalosporins, ciprofloxacin, and EtBr in BC adapted strains. Our results also displayed that increased sensitivity to acid, alkali, osmotic, ethanol, and oxidative stresses was observed in most strains after repeat exposure to BC. Rotating disinfecting agents may be necessary in maintaining high effectiveness of BC toward *L. monocytogenes*. From our results, ethanol and hydrogen peroxide appear to be the most appropriate candidates of alternative methods for disinfection.

## Author contributions

XJ, WG, and LS designed and supervised the study. TY, YZ, and SJ performed the experiments. TY analyzed data. TY and XJ drafted the manuscript.

### Conflict of interest statement

The authors declare that the research was conducted in the absence of any commercial or financial relationships that could be construed as a potential conflict of interest. The reviewer IR-B and handling Editor declared their shared affiliation.
