# Supplementary material for: Effect of Benzalkonium Chloride Adaptation on Sensitivity to Antimicrobial Agents and Tolerance to Environmental Stresses in Listeria monocytogenes
Source: Front Microbiol. 2018 Nov 28;9:2906. doi: 10.3389/fmicb.2018.02906 (PMC6279922; doi:10.3389/fmicb.2018.02906)
Supplement: Supplementary file 1 [file Table_1.DOCX]

TABLE S1 *L. monocytogenes* strains used in this study.

| Strain | Source | Serotype | PFGE pattern^a^ |
| --- | --- | --- | --- |
| HL11 | Cooked meat | 1/2a | P3 |
| HL15 | Raw pork meat | 1/2a | P3 |
| S7-48 | Food production environment | 1/2a | ND |
| HL35 | Raw pork meat | 1/2a | P4 |
| HL79 | Raw chicken meat | 1/2a | P5 |
| HL95 | Raw beef meat | 1/2a | P5 |
| HL38 | Raw chicken meat | 1/2a | P11 |
| HL39 | Vegetable | 1/2b | P10 |
| HL12 | Raw chicken meat | 1/2b | P8 |
| HL78 | Raw chicken meat | 1/2b | P2 |
| HL50 | Cooked meat | 1/2b | P12 |
| HL60 | Cooked meat | 1/2b | P12 |
| HL82 | Raw chicken meat | 1/2b | P14 |
| HL90 | Raw beef meat | 1/2c | P5 |
| HL17 | Raw chicken meat | 1/2c | P5 |
| HL26 | Raw pork meat | 1/2c | P5 |
| HL88 | Raw pork meat | 1/2c | P5 |
| HL06 | Raw pork meat | 1/2c | P4 |
| HL24 | Raw pork meat | 1/2c | P4 |
| HL28 | Raw chicken meat | 1/2c | P4 |
| S36-84 | Food production environment | 4b | ND |
| S45-86 | Raw chicken meat | 4b | ND |
| S51-88 | Raw chicken meat | 4b | ND |
| S15-90 | Food production environment | 4b | ND |
| S1-73 | Vegetable | 3a | ND |

^a^ ND, not determined.
