# Supplementary material for: Effect of Benzalkonium Chloride Adaptation on Sensitivity to Antimicrobial Agents and Tolerance to Environmental Stresses in Listeria monocytogenes
Source: Front Microbiol. 2018 Nov 28;9:2906. doi: 10.3389/fmicb.2018.02906 (PMC6279922; doi:10.3389/fmicb.2018.02906)
Supplement: Supplementary file 2 [file Table_2.DOCX]

TABLE S2 Primers used in this study.

| Primer^a^ | Sequence (5'-3')^b^ |
| --- | --- |
| Primers for RT-qPCR | |
| RT*mdrL*-F | TAAAGTGAAAGAACCGAAGA |
| RT*mdrL*-R | CAAACATAATCCCCAAGC |
| RT*lde*-F | GCGATGATTTTGATGGGA |
| RT*lde*-R | ACCGCTGCCGTTGATAGT |
| RT16S-F | GGGAGGCAGCAGTAGGGA |
| RT16S-R | CCGTCAAGGGACAAGCAG |
| Primers for gene deletion mutant strain construction | |
| *mdrL*-1 | *CG*GGATCCGTCCCTTGGTTCTGGCAT (*BamH*I) |
| *mdrL*-2 | GTTGTAAGGTAAAATGTGCTGGAATACAACTACACTTCCCTTTCC |
| *mdrL*-3 | GGAAAGGGAAGTGTAGTTGTATTCCAGCACATTTTACCTTACAAC |
| *mdrL*-4 | *CG*GAATTCTCCAATCATAAAGTTTCGTCAG (*EcoR*I) |
| *mdrL*-5 | TGTAAAGCAGCAGGAGTG |
| *mdrL*-6 | AAACGACGCTAATAACCAT |
| *lde*-1 | *GC*GTCGACTTTGGCACAGCATTAGGAT (*Sal*I) |
| *lde*-2 | GATAGAAGAATCTAGGTGGATTTTCTAATACAATTACCAGGAATAGGT |
| *lde*-3 | ACCTATTCCTGGTAATTGTATTAGAAAATCCACCTAGATTCTTCTATC |
| *lde*-4 | *CG*ACGCGTGACGATGGCTTGGTTCTG (*Mlu*I) |
| *lde*-5 | TCCGTTTCCGCAACATAG |
| *lde*-6 | GCACATTAGCCAATACCC |
| Primers for complementation | |
| *mdrL*-7 | *CC*GAGCTCTATCATACAATAGCACCTCTG (*Sac*I) |
| *mdrL*-8 | *CG*GGATCCCCAGGTTACTCAGTCTCTTG (*BamH*I) |

^a^ Primers for RT-qPCR and the construction of the deletion mutant and complemented strains were designed using Primer Premier 5.0.

^b^ Restriction sites are underlined.
