# Supplementary material for: Effect of Benzalkonium Chloride Adaptation on Sensitivity to Antimicrobial Agents and Tolerance to Environmental Stresses in Listeria monocytogenes
Source: Front Microbiol. 2018 Nov 28;9:2906. doi: 10.3389/fmicb.2018.02906 (PMC6279922; doi:10.3389/fmicb.2018.02906)
Supplement: Supplementary file 3 [file Table_3.DOCX]

TABLE S3 Area under curve (AUC) values of the wild-type and BC adapted strains of *L. monocytogenes*.

| Strain | AUC±SD^a^ | | | | | | | | | |
| --- | --- | --- | --- | --- | --- | --- | --- | --- | --- | --- |
|  | pH 5.5 | | pH 9.5 | | 6% NaCl | | 3.5% ethanol | | 1 mM H_2_O_2_ | |
|  | Original | Adapted | Original | Adapted | Original | Adapted | Original | Adapted | Original | Adapted |
| HL11 | 12.49±0.14 | 11.37±0.45* | 15.76±0.29 | 15.01±0.54 | 18.67±0.46 | 13.34±0.56* | 20.48±0.52 | 18.38±0.98** | 12.61±0.29 | 11.34±0.35 |
| HL15 | 11.06±0.46 | 11.04±0.76 | 14.27±0.42 | 13.94±0.54* | 17.18±0.54 | 16.31±0.72 | 20.81±0.51 | 20.53±0.67 | 11.49±0.55 | 11.32±0.37 |
| S7-48 | 11.10±0.83 | 10.02±0.55 | 15.72±0.43 | 14.39±0.26 | 17.23±0.74 | 15.02±1.01 | 20.31±0.62 | 18.95±0.72 | 13.92±0.30 | 11.02±0.56 |
| HL35 | 8.30±0.55 | 7.83±0.27 | 15.62±0.41 | 13.92±0.98 | 19.65±0.23 | 16.21±0.28 | 19.83±0.63 | 17.24±0.76* | 13.82±29 | 10.93±0.75** |
| HL79 | 12.13±0.19 | 11.81±0.27 | 15.79±0.38 | 13.81±0.45 | 19.92±0.72 | 15.99±0.96 | 20.44±0.27 | 17.62±0.79 | 13.86±0.27 | 10.95±0.72 |
| HL95 | 12.09±0.28 | 11.72±0.31 | 14.28±0.29 | 12.46±0.77 | 18.48±0.48 | 15.23±0.87 | 21.21±0.58 | 18.92±0.91 | 12.85±0.28 | 10.81±0.18 |
| HL38 | 11.24±0.23 | 10.68±0.59 | 14.40±0.16 | 12.51±0.78 | 17.91±0.99 | 15.02±0.81 | 20.49±0.24 | 18.24±0.89 | 13.52±0.81 | 9.52±0.23* |
| HL39 | 12.27±0.14 | 12.21±0.67 | 15.56±0.17 | 14.79±0.28 | 17.83±0.94 | 16.04±0.67 | 20.17±0.63 | 18.22±0.47 | 13.85±0.19 | 12.87±0.59 |
| HL12 | 7.89±0.24 | 6.89±0.34 | 11.82±0.33 | 9.91±0.23** | 18.71±0.75 | 15.82±0.36 | 19.61±0.67 | 18.00±0.72 | 13.46±0.29 | 10.28±0.60 |
| HL78 | 7.24±0.19 | 6.58±0.33 | 11.90±0.26 | 10.02±0.33 | 18.82±0.77 | 15.84±0.71 | 20.19±0.79 | 18.11±0.92 | 12.63±0.63 | 9.03±0.62 |
| HL50 | **7.19±0.38** | **5.25±0.69*** | 11.94±0.33 | 11.08±0.35 | 17.23±0.38 | 15.90±0.34 | 19.40±0.90 | 17.20±0.43 | 12.69±0.33 | 9.95±0.49 |
| HL60 | 7.40±0.44 | 7.07±0.78 | 10.30±0.45 | 9.97±0.76 | 19.23±0.27 | 17.03±0.78 | 19.68±0.94 | 19.64±0.84* | 13.23±0.25 | 6.91±0.99 |
| HL82 | 7.12±0.43 | 6.01±0.56 | 10.75±0.56 | 10.68±0.77 | 19.25±0.21 | 15.02±0.45 | 19.19±0.81 | 12.95±0.79 | 13.40±0.22 | 10.57±0.75* |
| HL90 | 10.73±0.56 | 10.02±0.77** | 15.28±0.24 | 14.89±0.62* | **20.02±0.50** | **10.32±0.38*** | **20.05±0.45** | **9.93±0.56*** | **13.18±0.29** | **2.43±0.74**** |
| HL17 | 7.31±0.49 | 6.62±0.45 | 9.43±0.38 | 8.87±0.57 | 19.78±0.64 | 15.58±0.55 | 17.59±0.72 | 14.30±0.47 | 13.92±0.25 | 9.89±0.93 |
| HL26 | 7.20±0.44 | 6.87±0.47 | 10.15±0.65 | 7.61±0.36 | 19.50±0.98 | 15.88±0.81** | 19.65±0.76 | 15.49±0.43 | 13.99±0.16 | 12.48±0.61 |
| HL88 | 10.73±0.44 | 9.92±0.44 | 14.62±0.95 | 12.71±0.58 | 19.73±0.76 | 15.67±0.59 | 19.54±0.44 | 17.23±0.37* | 11.71±0.56 | 7.99±0.66* |
| HL06 | 7.92±0.35 | 6.82±0.72 | 14.39±0.91 | 13.02±0.52* | 19.52±0.49 | 15.90±0.87 | 19.52±0.42 | 17.90±0.45 | 12.93±0.52 | 9.61±0.78 |
| HL24 | 7.07±0.57 | 5.73±0.67 | 7.52±0.44 | 7.05±0.64 | 19.39±0.43 | 13.54±0.41 | 19.34±0.44 | 10.02±0.55 | 12.76±0.35 | 5.88±0.82 |
| HL28 | 10.45±0.38 | 8.59±0.49* | 13.04±0.51 | 10.48±0.35 | 19.24±0.55 | 15.18±0.88** | 20.83±0.96 | 17.98±0.89* | 13.52±0.58 | 9.60±0.46 |
| S36-84 | 7.16±0.27 | 7.01±0.72 | 13.52±0.53 | 11.23±0.67 | 18.81±0.47 | 15.49±0.92 | 20.27±0.95 | 17.92±0.98 | 13.64±0.67 | 12.84±0.44 |
| S45-86 | 7.82±0.47 | 6.74±0.71 | 13.49±0.76 | 11.05±0.66 | 18.98±0.44 | 15.51±0.36* | 20.71±0.71 | 17.51±0.59* | 14.40±0.91 | 13.59±0.38 |
| S51-88 | 6.74±0.38 | 7.22±0.65 | 11.76±0.56 | 6.69±0.89 | 18.96±0.52 | 17.76±0.42 | 20.99±0.73 | 17.48±0.68 | 14.23±0.31 | 13.10±0.45 |
| S15-90 | 7.39±0.59 | 7.35±0.73 | 11.95±0.55 | 7.69±0.92 | 19.11±0.56 | 15.90±0.44 | 20.46±0.82 | 17.84±0.95 | 14.27±0.35 | 12.86±0.92* |
| S1-73 | 6.51±0.29 | 6.97±0.77 | **10.44±0.48** | **4.94±0.37*** | 17.96±0.56 | 17.38±0.46 | 17.93±0.78 | 17.58±0.87 | 12.84±0.57 | 11.83±0.42 |

^a^ Statistically significant values are indicated by asterisks (**, *P*<0.001; *, *P*<0.01) (*P* values were obtained using 2-tailed *t*-test). The largest decrease between original and adapted strains were marked in bold.
