# Supplementary material for: Effect of Benzalkonium Chloride Adaptation on Sensitivity to Antimicrobial Agents and Tolerance to Environmental Stresses in Listeria monocytogenes
Source: Front Microbiol. 2018 Nov 28;9:2906. doi: 10.3389/fmicb.2018.02906 (PMC6279922; doi:10.3389/fmicb.2018.02906)
Supplement: Supplementary file 4 [file Table_4.DOCX]

TABLE S4 Mean maximum growth rates of the wild-type and BC adapted strains of *L. monocytogenes*.

| Strain | Maximum growth rate ±SD (OD_600_ units/h)^a^ | | | | | | | | | |
| --- | --- | --- | --- | --- | --- | --- | --- | --- | --- | --- |
|  | pH 5.5 | | pH 9.5 | | 6% NaCl | | 3.5% ethanol | | 1 mM H_2_O_2_ | |
|  | Original | Adapted | Original | Adapted | Original | Adapted | Original | Adapted | Original | Adapted |
| HL11 | **0.154±0.008** | **0.096±0.004**** | 0.236±0.007 | 0.196±0.004* | 0.078±0.0005 | 0.059±0.0006** | 0.047±0.002 | 0.045±0.002 | 0.189±0.003 | 0.155±0.004** |
| HL15 | 0.107±0.002 | 0.106±0.003 | 0.251±0.007 | 0.238±0.005 | 0.067±0.0008 | 0.064±0.0007 | 0.052±0.001 | 0.048±0.001* | 0.158±0.002 | 0.135±0.003** |
| S7-48 | 0.146±0.006 | 0.138±0.004 | 0.234±0.002 | 0.211±0.005* | 0.069±0.0012 | 0.057±0.0025* | 0.055±0.001 | 0.045±0.001* | 0.231±0.005 | 0.200±0.008* |
| HL35 | 0.152±0.009 | 0.097±0.002** | 0.217±0.002 | 0.203±0.006* | 0.079±0.0018 | 0.060±0.0012** | 0.050±0.001 | 0.046±0.001 | 0.185±0.005 | 0.158±0.004** |
| HL79 | 0.144±0.008 | 0.135±0.004 | 0.231±0.007 | 0.197±0.004* | 0.064±0.0017 | 0.059±0.0018* | 0.054±0.002 | 0.047±0.001* | 0.135±0.003 | 0.124±0.003** |
| HL95 | 0.107±0.004 | 0.104±0.004 | 0.127±0.003 | 0.126±0.004 | 0.067±0.0003 | 0.064±0.0006 | 0.051±0.001 | 0.049±0.001* | 0.225±0.007 | 0.193±0.006* |
| HL38 | 0.105±0.003 | 0.104±0.004 | 0.251±0.009 | 0.247±0.008 | 0.063±0.0012 | 0.059±0.0011 | 0.053±0.001 | 0.031±0.000** | 0.160±0.006 | 0.143±0.005** |
| HL39 | 0.155±0.003 | 0.140±0.003* | 0.275±0.006 | 0.230±0.005** | 0.062±0.0015 | 0.058±0.0014 | 0.054±0.001 | 0.031±0.001** | 0.148±0.004 | 0.202±0.003** |
| HL12 | 0.152±0.007 | 0.143±0.008* | 0.132±0.002 | 0.123±0.003* | 0.076±0.0002 | 0.049±0.0001** | 0.054±0.001 | 0.042±0.001* | 0.197±0.006 | 0.142±0.005** |
| HL78 | 0.082±0.002 | 0.074±0.001* | 0.140±0.003 | 0.127±0.002* | 0.071±0.0011 | 0.064±0.0012* | 0.061±0.001 | 0.056±0.001* | 0.204±0.007 | 0.152±0.005** |
| HL50 | 0.076±0.002 | 0.050±0.004* | 0.134±0.002 | 0.124±0.001* | 0.075±0.0017 | 0.050±0.0014** | 0.052±0.002 | 0.044±0.001* | 0.132±0.001 | 0.098±0.003** |
| HL60 | 0.080±0.002 | 0.074±0.001* | 0.132±0.005 | 0.122±0.004 | 0.069±0.0015 | 0.056±0.0042* | 0.063±0.002 | 0.029±0.001** | 0.151±0.003 | 0.135±0.003* |
| HL82 | 0.069±0.000 | 0.053±0.002* | 0.139±0.001 | 0.125±0.004* | 0.062±0.0005 | 0.054±0.0010** | 0.055±0.001 | 0.053±0.001 | 0.149±0.002 | 0.111±0.001** |
| HL90 | 0.066±0.001 | 0.052±0.001** | 0.205±0.007 | 0.175±0.007** | 0.066±0.0027 | 0.028±0.0018** | 0.047±0.002 | 0.022±0.001** | **0.127±0.002** | **0.059±0.001**** |
| HL17 | 0.066±0.000 | 0.052±0.004* | 0.127±0.001 | 0.101±0.001** | 0.071±0.0008 | 0.023±0.0021** | 0.034±0.001 | 0.026±0.002* | 0.132±0.003 | 0.108±0.001** |
| HL26 | 0.052±0.001 | 0.070±0.002** | 0.159±0.002 | 0.135±0.001** | 0.070±0.0017 | 0.067±0.0017 | 0.042±0.001 | 0.030±0.002* | 0.150±0.001 | 0.197±0.002** |
| HL88 | 0.063±0.001 | 0.044±0.000** | 0.134±0.003 | 0.128±0.005 | 0.078±0.0011 | 0.074±0.0009 | 0.036±0.001 | 0.029±0.000* | 0.200±0.006 | 0.149±0.005** |
| HL06 | 0.067±0.001 | 0.054±0.000* | 0.127±0.003 | 0.102±0.002** | 0.075±0.0013 | 0.069±0.0006* | 0.042±0.001 | 0.031±0.001** | 0.204±0.007 | 0.131±0.004** |
| HL24 | 0.063±0.000 | 0.043±0.001** | 0.131±0.003 | 0.099±0.003** | **0.078±0.0026** | **0.025±0.0006**** | **0.039±0.001** | **0.016±0.001**** | 0.143±0.004 | 0.109±0.002** |
| HL28 | 0.100±0.001 | 0.074±0.001** | **0.219±0.006** | **0.160±0.007**** | 0.065±0.0034 | 0.024±0.0011** | 0.058±0.000 | 0.035±0.002** | 0.147±0.004 | 0.097±0.001** |
| S36-84 | 0.072±0.002 | 0.058±0.001** | 0.170±0.003 | 0.158±0.004* | 0.076±0.0009 | 0.070±0.0008* | 0.049±0.001 | 0.030±0.000** | 0.211±0.007 | 0.129±0.005** |
| S45-86 | 0.078±0.002 | 0.065±0.001* | 0.171±0.004 | 0.165±0.003* | 0.069±0.0007 | 0.057±0.0011** | 0.045±0.001 | 0.031±0.001** | 0.200±0.006 | 0.132±0.006** |
| S51-88 | 0.058±0.000 | 0.070±0.000** | 0.175±0.004 | 0.163±0.002* | 0.067±0.0011 | 0.052±0.0012** | 0.051±0.001 | 0.034±0.001** | 0.129±0.002 | 0.216±0.003** |
| S15-90 | 0.064±0.003 | 0.078±0.002* | 0.169±0.002 | 0.161±0.002* | 0.063±0.0014 | 0.051±0.0012** | 0.053±0.001 | 0.051±0.001 | 0.134±0.002 | 0.209±0.007** |
| S1-73 | 0.057±0.001 | 0.054±0.003 | 0.165±0.006 | 0.167±0.007 | 0.066±0.0004 | 0.065±0.0004 | 0.032±0.000 | 0.027±0.000** | 0.208±0.003 | 0.137±0.003** |

^a^ Statistically significant values are indicated by asterisks (**, *P*<0.001; *, *P*<0.01) (*P* values were obtained using 2-tailed *t*-test). The largest decrease between original and adapted strains were marked in bold.
